# Examining Feasibility, Acceptability, and Exploratory Pre–Post Changes Following Faculty-Delivered Mindfulness-Based Attention Training in Pediatric Critical Care

**DOI:** 10.3390/healthcare14213620

**Published:** 2026-10-26

**Authors:** Julie Katia Morin-Major, Lauren Yagiela, Dennis Leung, Mohammad Bilal Khan, Malena M. Price, Scott L. Rogers, Amishi P. Jha

**Affiliations:** 1 Division of Critical Care, Department of Pediatrics, Children’s Hospital of Michigan, Detroit, MI 48201, USA; 2 Department of Pediatrics, College of Medicine, Central Michigan University, Mt. Pleasant, MI 48859, USA; 3 Division of Critical Care, Loma Linda Children’s Hospital, Loma Linda, CA 92354, USA; 4 Department of Psychology, University of Miami, Coral Gables, FL 33146, USA; 5 Department of Psychiatry, Massachusetts General Hospital, Boston, MA 02114, USA; 6 Department of Psychiatry, Harvard Medical School, Boston, MA 02115, USA; 7 School of Law, University of Miami, Coral Gables, FL 33146, USA

**Keywords:** attention, burnout, cortisol, Graduate Medical Education, mindfulness, pediatric critical care, physician well-being, stress

## Abstract

Background: Pediatric critical care medicine (PCCM) fellows experience substantial psychological stress and cognitive demands, yet evidence-based interventions to support their well-being remain limited. As a preliminary step to address this gap, we evaluated the feasibility and acceptability of faculty-delivered Mindfulness-Based Attention Training (MBAT) within a PCCM fellowship. Methods: In this single-arm pilot study, eight PCCM fellows completed a faculty-delivered 4-week (8 h) MBAT program. Primary outcomes were feasibility and acceptability. Feasibility was assessed across three domains: intervention delivery and attendance, independent mindfulness practice, and completion of planned assessments and biospecimen collection. Acceptability was assessed by participant ratings of motivation, application of MBAT skills to daily life, and willingness to recommend the course to colleagues. Exploratory pre–post outcomes included participant-reported psychological measures, Sustained Attention to Response Task (SART) performance, and hair cortisol concentration. Results: All planned MBAT sessions were delivered; seven participants (87.5%) attended all four sessions, six (75.0%) practiced independently 1–2 times per week, and all participants completed planned assessments. For acceptability, 75.0% agreed that they applied MBAT skills to daily life, 62.5% were motivated to participate, and 87.5% would recommend the course to colleagues. Exploratory pre–post analyses showed higher mean positive affect and lower mean burnout, perceived stress, anxiety and depressive symptoms, SART reaction time and error counts, and hair cortisol concentration. Conclusions: Faculty-delivered MBAT was feasible to deliver and attend, with complete study assessment and biospecimen collection, and was generally well accepted within a PCCM fellowship. However, independent participant practice was limited, representing a key feasibility and implementation challenge that future investigations may address through asynchronous, self-paced program content.

## 1. Introduction

During medical training, physicians experience substantial psychological stress alongside sustained cognitive demands [1]. Trainees must acquire complex clinical knowledge, develop new procedural skills, communicate effectively with patients and families, and make consequential decisions under pressure. Meeting these demands requires the capacity to sustain attention, manage cognitive and emotional strain, and recognize when fatigue may compromise performance [2,3,4]. Yet traditional medical training has devoted limited attention to developing these capacities. In 2017, the Accreditation Council for Graduate Medical Education (ACGME) revised its program requirements to place greater emphasis on physician well-being. However, relatively few interventions have been rigorously evaluated, and fewer still have incorporated objective measures of cognitive performance or physiological stress.

The pediatric critical care medicine (PCCM) fellowship represents an especially cognitively and emotionally demanding training environment. Fellows must continuously integrate complex clinical information across multiple critically ill patients while responding to frequent interruptions, coordinating with multidisciplinary teams, communicating with families, and making time-sensitive decisions for which lapses in attention may have serious consequences. These cognitive demands are compounded by repeated exposure to critically ill children, end-of-life care, and moral distress, each of which may contribute to burnout [5]. National data indicate that burnout is highly prevalent among PCCM fellows and is associated with sleep deprivation, lower career satisfaction, workplace conflict, and reduced social support [6]. Pediatric residents similarly report increased emotional exhaustion, depersonalization, and depressive symptoms following pediatric intensive care unit rotations [7]. These findings underscore the need for interventions that support both attentional functioning and psychological well-being in this setting.

From a self-regulation perspective, these demands require fellows to continuously monitor and regulate attention, cognition, and emotional responses in the service of clinical goals. High cognitive workload, frequent interruptions, fatigue, and emotional stress may tax these regulatory capacities, increasing vulnerability to attentional lapses and reducing the cognitive resources available for complex decision-making. Mindfulness-based attention training (MBAT) [8] is theoretically relevant in this context because it explicitly trains participants to monitor the current focus of attention, recognize mind wandering and distraction, and intentionally redirect attention to task-relevant experience. Thus, MBAT may be particularly applicable to high-demand clinical training environments such as PCCM, where effective performance depends on maintaining and flexibly regulating attention amid competing cognitive and emotional demands. This rationale does not presume that MBAT is uniquely suited to PCCM; rather, the intensity and convergence of these demands make PCCM an important setting in which to examine the feasibility and acceptability of an attention-focused self-regulatory intervention.

Mindfulness-based interventions have shown potential to reduce burnout and perceived stress among physicians and trainees, although findings remain mixed. A systematic review and meta-analysis found reductions in burnout and stress but rated the overall quality of evidence as low to very low [9], while a large multicenter randomized trial among pediatric interns found no significant benefit on emotional exhaustion, burnout, empathy, or mindfulness [10]. Most published studies have focused on medical students or resident physicians, leaving PCCM fellows particularly understudied [11,12]. Existing studies have also relied primarily on self-reported outcomes, providing limited evidence about effects on cognitive performance or physiological stress; notably, a randomized trial in resident physicians found no significant effect on hair cortisol [13]. Combining self-reported metrics with behavioral measures of attention and physiological markers of stress may provide a more complete account of how mindfulness training may affect trainee well-being and performance. Before adequately powered randomized trials can evaluate these outcomes, the feasibility and acceptability of delivering such training within the demanding context of PCCM fellowship must first be established.

Although mindfulness training programs are increasingly common in medical education, successful implementation in demanding clinical settings requires programs that are both time-efficient and scalable. A train-the-trainer model may be especially valuable because it enables context-familiar faculty to deliver the curriculum and connect its content to the specific demands trainees face. Importantly, mindfulness-based interventions encompass heterogeneous approaches that vary in curriculum, duration, intensity, and mode of delivery; therefore, findings from the broader mindfulness literature may not be directly generalizable to individual programs. Within this broader landscape, MBAT is a distinct, standardized four-week, eight-hour program developed for high-stress professional settings that incorporates a train-the-trainer pathway [8]. MBAT has been evaluated primarily in military and other high-demand occupational cohorts, with studies examining cognitive, psychological, social, and performance-related outcomes [8,14,15,16,17,18]. More recently, MBAT was adapted for medical education, with physician faculty trained to deliver a version contextualized for medical students. Voluntary program evaluations indicated that students perceived benefits for attention, psychological health, interpersonal functioning, and professional development and viewed the faculty-delivered program as relevant to medical training [19]. However, MBAT has not yet been formally evaluated among graduate medical trainees or within PCCM fellowship.

The primary objective of this pilot study was therefore to evaluate the feasibility and acceptability of implementing faculty-delivered MBAT within a PCCM fellowship. We examined barriers to participation and assessed whether fellowship faculty could integrate the program into an already demanding training environment. Secondary exploratory objectives were to examine pre–post changes in participant-reported measures of positive affect, burnout, perceived stress, anxiety and depressive symptoms, objective measures of attention, and hair cortisol concentration. We hypothesized that faculty-delivered MBAT would be feasible and acceptable within a PCCM fellowship; exploratory pre–post outcomes were examined descriptively to inform the design of future controlled studies.

## 2. Materials & Methods

### 2.1. Design, Setting, and Participants

A single-arm pilot study was conducted to evaluate the feasibility and acceptability of implementing faculty-delivered MBAT within a PCCM fellowship. Feasibility and acceptability of implementing MBAT were the primary outcomes. Participant-reported psychological measures, objectively measured attention, and hair cortisol concentration were exploratory outcomes. The study took place at Children’s Hospital of Michigan in Detroit, Michigan, between March and June 2025. All eight PCCM fellows at the institution were invited to participate. Participation in the research study involved only the evaluation and data-collection procedures and did not determine eligibility for, access to, or assignment to the MBAT; MBAT was offered to the fellowship cohort independently of research participation. The Central Michigan University Institutional Review Board approved the protocol, and all participants provided written informed consent before enrollment. The study was not prospectively registered in a clinical trial registry. Study data were assigned unique identifying numbers and de-identified before analysis.

### 2.2. Intervention Description

MBAT is a standardized, four-week, eight-hour mindfulness curriculum developed for professionals working in high-demand, high-stress settings [8,19]. The program is designed to strengthen attention and support well-being and performance under demanding professional and personal conditions. It cultivates mindfulness, defined as “a mental mode characterized by attention to present-moment experience without conceptual elaboration or emotional reactivity” [20], through didactic instruction, guided reflection, group discussion, and formal mindfulness practice.

In the present study, two PCCM faculty members delivered MBAT to PCCM fellows after completing a 16-week hybrid train-the-trainer practicum led by the program’s co-creators. The practicum included approximately two hours of virtual training per week and one extended in-person session. It prepared the faculty members to deliver the standardized curriculum while contextualizing its content for PCCM fellowship.

During delivery to the PCCM fellows, each of the four two-hour sessions followed a standardized structure and addressed two core themes, with approximately one hour devoted to each. For each theme, the trainer introduced the topic, guided participants through reflective prompts and group discussion, led a 15 min mindfulness practice, and facilitated a post-practice discussion designed to deepen participants’ understanding of their practice experience. In addition to participating in the four live sessions, fellows were asked to engage in 15 min mindfulness exercises assigned as homework for each week, at least four days per week. Formal intervention fidelity was not prospectively assessed.

### 2.3. Procedure

One week prior to the start of the MBAT program (T1), participants completed a 30 min baseline assessment consisting of self-report questionnaire including the positive affect scale, the burnout assessment tool, the perceived stress scale and the patient health questionnaire as well as an objective measure of attention administered on a designated research computer using the Inquisit 7 platform (Millisecond Software, Seattle, WA, USA, 2024). A hair sample was also collected to assess hair cortisol concentration, a biomarker of chronic stress. Five weeks later, approximately one week following completion of the MBAT program, participants completed a second assessment (T2) consisting of the same self-report questionnaires, objective measure of attention, and a post-intervention feedback survey assessing feasibility and acceptability. A second hair sample was collected at the post-intervention assessment. Assessments were completed privately and outside of the MBAT sessions on a designated research computer. Participants generated their own four-digit study code, which was used to link T1 and T2 data without linking responses to participant identity. The faculty members who delivered MBAT were not involved in data collection and did not have access to participant responses. Participants did not receive financial compensation for study participation.

### 2.4. Measures

In accordance with the first study objective, feasibility and acceptability were assessed descriptively using an investigator-developed feedback survey and completion of planned assessments (self-report questionnaires, objective measure of attention). Feasibility was evaluated across three domains: (1) feasibility of delivering the intervention and participants’ ability to attend scheduled MBAT sessions, (2) feasibility of engaging in mindfulness practice outside the scheduled training sessions, and (3) feasibility of completing planned study assessments and biospecimen collection.

For feasibility domain #1, each of the four MBAT sessions was offered at two separate times to accommodate fellows’ clinical schedules, and participants were recommended to attend one of each session. Participants reported the number of sessions attended and any barriers to attendance. For feasibility domain #2, participants reported the frequency of independent mindfulness practice outside scheduled sessions (not at all, 1–2 times/week, 3–4 times/week, or ≥5 times/week), typical practice duration (<5 min, 5–10 min, 11–20 min, or >20 min), and barriers to independent practice. Participants also rated the overall quality of their mindfulness practice over the preceding month on a 5-point scale ranging from very low to very high. Brief free-text responses to the two barrier questions were independently reviewed by two authors who were not involved in delivering MBAT and summarized using descriptive qualitative content analysis, with conceptually similar responses grouped into categories. Because participants could report more than one barrier, categories were not mutually exclusive. Adverse effects were not systematically assessed.

For feasibility domain #3, data-collection feasibility was assessed by completion of the planned participant-reported psychological measures, Sustained Attention to Response Task (SART), and hair cortisol collection at both T1 and T2.

Acceptability was evaluated separately using three 5-point Likert-scale items assessing participants’ motivation to participate in MBAT, application of MBAT knowledge and skills to daily life, and willingness to recommend a similar course to colleagues. No a priori feasibility, acceptability, or progression thresholds were prespecified; these outcomes were characterized descriptively within each assessed domain.

As part of the second study objective, participants completed a series of self-reported psychological measures, an objective measure of attention, and a measure of hair cortisol concentration as a biomarker of chronic stress which are described in more detail below.

Positive affect subscale of the Positive and Negative Affect Schedule (PANAS) [21]. Positive affect was assessed using the 10-item positive affect subscale of the PANAS. The subscale yields a total score ranging from 10 to 50, with higher scores indicating greater positive affect. The negative affect subscale was not administered.

Burnout Assessment Tool-12 (BAT-12) [22,23]. The BAT-12 assesses four core dimensions of burnout: exhaustion, mental distance, emotional impairment, and cognitive impairment. Items are rated on a 5-point frequency scale ranging from 1 (“never”) to 5 (“always”) and averaged to yield an overall mean score, with higher scores indicating greater burnout symptoms.

Perceived Stress Scale (PSS) [24]. The PSS is a 10-item scale. This instrument assesses how individuals perceive situations of their daily lives as stressful, unpredictable, or overwhelming. The questionnaire is scored to generate a total score between 0 and 40, with higher scores indicating greater perceived stress.

Patient Health Questionnaire-4 (PHQ-4) [25]. The PHQ-4 was used to assess anxiety and depressive symptoms over the preceding two weeks. It consists of four items, including two assessing depressive symptoms and two assessing anxiety symptoms. Each item is scored from 0 to 3, yielding a total score ranging from 0 to 12, with higher scores indicating greater anxiety and depressive symptom burden.

Sustained Attention to Response Task (SART) [26]. The SART is a validated computerized go/no-go paradigm to measure sustained attention and lapse of attention over time. Participants were presented with a continuous stream of single digits and instructed to withhold a response when presented with the digit 3 (No-Go target). Stimuli were presented briefly and at fixed intervals for 7 min which favored an automatic response tendency. A commission (failure to withhold a no-go target) indicated a lapse in attentional awareness while variable response time and omission errors provided a measure of sustained attention performance. The SART was scored to obtain the reaction time and the number of omissions and commissions for each participant at both T1 and T2.

Hair cortisol concentration. Hair cortisol concentration was collected as a biomarker of chronic stress. Hair samples were collected from the posterior vertex of the scalp by a study investigator with prior training in hair sampling. A small lock of hair was cut as close to the scalp as possible, with the proximal (root) end identified. Hair coloring or other chemical treatment was not systematically recorded. For analysis, the proximal 3 cm segment was used, representing approximately three months of hair growth [27,28]. Approximately 25 mg of hair was washed twice with 2.5 mL isopropanol for 3 min and allowed to dry overnight. Cortisol was extracted by incubating the hair in 1.5 mL methanol for 24 h with slow rotation, based on previously described methods [29]. Samples were then centrifuged for 2 min at 10,000 rpm, and 1 mL of supernatant was transferred to a fresh vial and evaporated under nitrogen at 60 °C. Dried extracts were reconstituted in 0.4 mL phosphate buffer, and cortisol concentrations were determined using a commercially available chemiluminescence immunoassay (IBL-International, Hamburg, Germany). Samples were analyzed in duplicate on a single assay plate and assay concentrations were reported in µg/dL. These were converted to pg/mg of hair using each sample’s recorded hair mass, the reconstitution volume, and the proportion of methanol extract analyzed. The reportable assay range was 0.012–3.2 µg/dL, and the assay CV for the plate was 2.71%.

### 2.5. Statistical Analysis

For this study’s first objective, feasibility and acceptability outcomes were summarized descriptively using counts and percentages. For the second study objective, exploratory pre–post outcomes were analyzed using an estimation-focused approach. For each outcome, T1 and T2 values were summarized using means and standard deviations, and the mean paired change was calculated as T2–T1. 95% confidence intervals for mean paired changes were calculated using Student’s t-distribution based on the paired difference scores. Standardized effect estimates were calculated using Cohen’s d_z_, defined as the mean paired change divided by the standard deviation of the paired difference scores. 95% confidence intervals for Cohen’s d_z_ were derived from the noncentral t distribution. Given the small sample size and exploratory nature of these secondary outcomes, no formal null-hypothesis significance testing was performed and corrections for multiple comparisons were not applied. Instead, interpretation emphasized the magnitude and uncertainty of the estimates (mean paired change and effect size, with 95% confidence intervals) and individual participant trajectories. For highly skewed data, T1 and T2 medians and interquartile ranges, along with median within-participant changes, were also reported to supplement the mean-based summaries. Statistical analyses were conducted by the first author, who was not involved in delivery of the MBAT intervention. The MBAT developers who were study coauthors did not have access to participant-level data and were not involved in data handling or conducting statistical analyses.

## 3. Results

### 3.1. Demographics

All eight eligible PCCM fellows were invited to participate and enrolled in the study between March and June 2025, corresponding to a recruitment rate of 100% (8/8). All participants completed the baseline (T1) and post-intervention (T2) assessments. The sample consisted of 6 females and 2 males. Two participants (25%) were between 20 and 29 years of age, while six (75%) were between 30 and 39 years of age. At the time of enrollment, three participants were in their first year of fellowship, two were in their second year, and three were in their final year of PCCM fellowship training.

### 3.2. Feasibility and Acceptability

All eight planned MBAT session offerings were delivered as intended. Seven participants (87.5%) attended all four sessions, while one participant (12.5%) attended three sessions because of a vacation-related scheduling conflict (Table 1). Seven participants (87.5%) reported engaging in independent mindfulness practice outside the scheduled MBAT sessions. Six (75.0%) reported practicing 1–2 times per week, one (12.5%) reported practicing 3–4 times per week, and one (12.5%) reported no independent practice. Among participants who practiced independently (*n* = 7), two (28.6%) typically practiced for <5 min, two (28.6%) for 5–10 min, and three (42.9%) for 11–20 min.

Five participants (62.5%) reported at least one barrier to independent practice, while three (37.5%) reported none. Difficulty remembering to practice was reported by four participants (50.0%), limited time by two (25.0%), motivation or difficulty committing to practice by two (25.0%), and fatigue or exhaustion associated with clinical service by one (12.5%). Participants rated the overall quality of their mindfulness practice over the preceding month as low (3/8, 37.5%), moderate (4/8, 50.0%), or high (1/8, 12.5%) (Table 1).

All planned study assessments were completed by all eight participants at both T1 and T2, including the participant-reported psychological measures, SART assessments, and hair cortisol collection, and all participants completed the post-intervention survey. Regarding acceptability, six participants (75.0%) agreed that they applied MBAT knowledge and skills to their daily lives, five (62.5%) agreed or strongly agreed that they were motivated to participate in the training, and seven (87.5%) agreed or strongly agreed that they would recommend a similar mindfulness course to colleagues (Table 1).

### 3.3. Exploratory Pre–Post Outcomes

**Figure 1:**
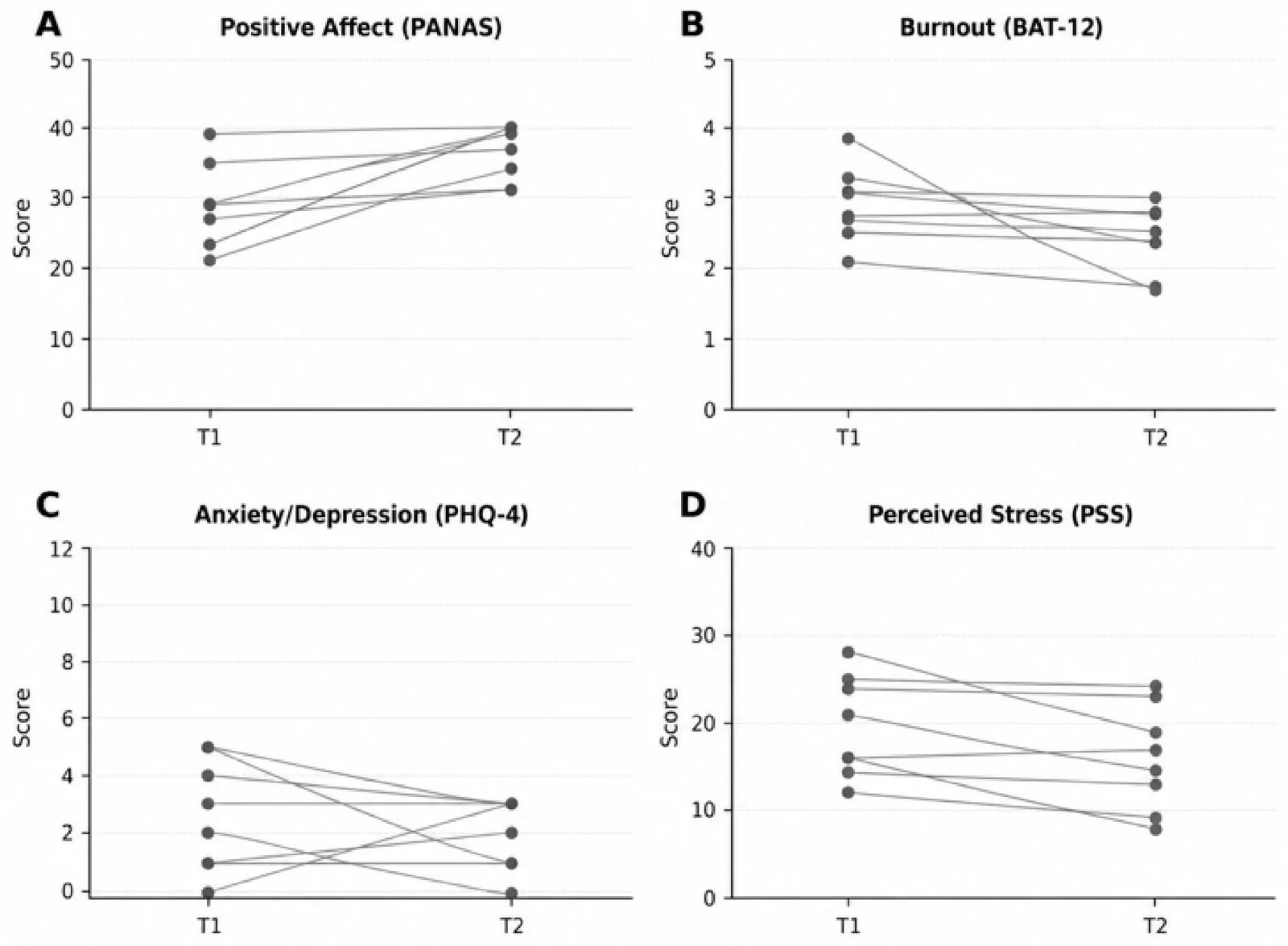
Individual Participant Changes from baseline (T1) to post-intervention (T2): (**A**) positive affect; (**B**) burnout; (**C**) perceived stress; (**D**) anxiety and depressive symptoms. Lines connect paired observations for each participant.

**Figure 2:**
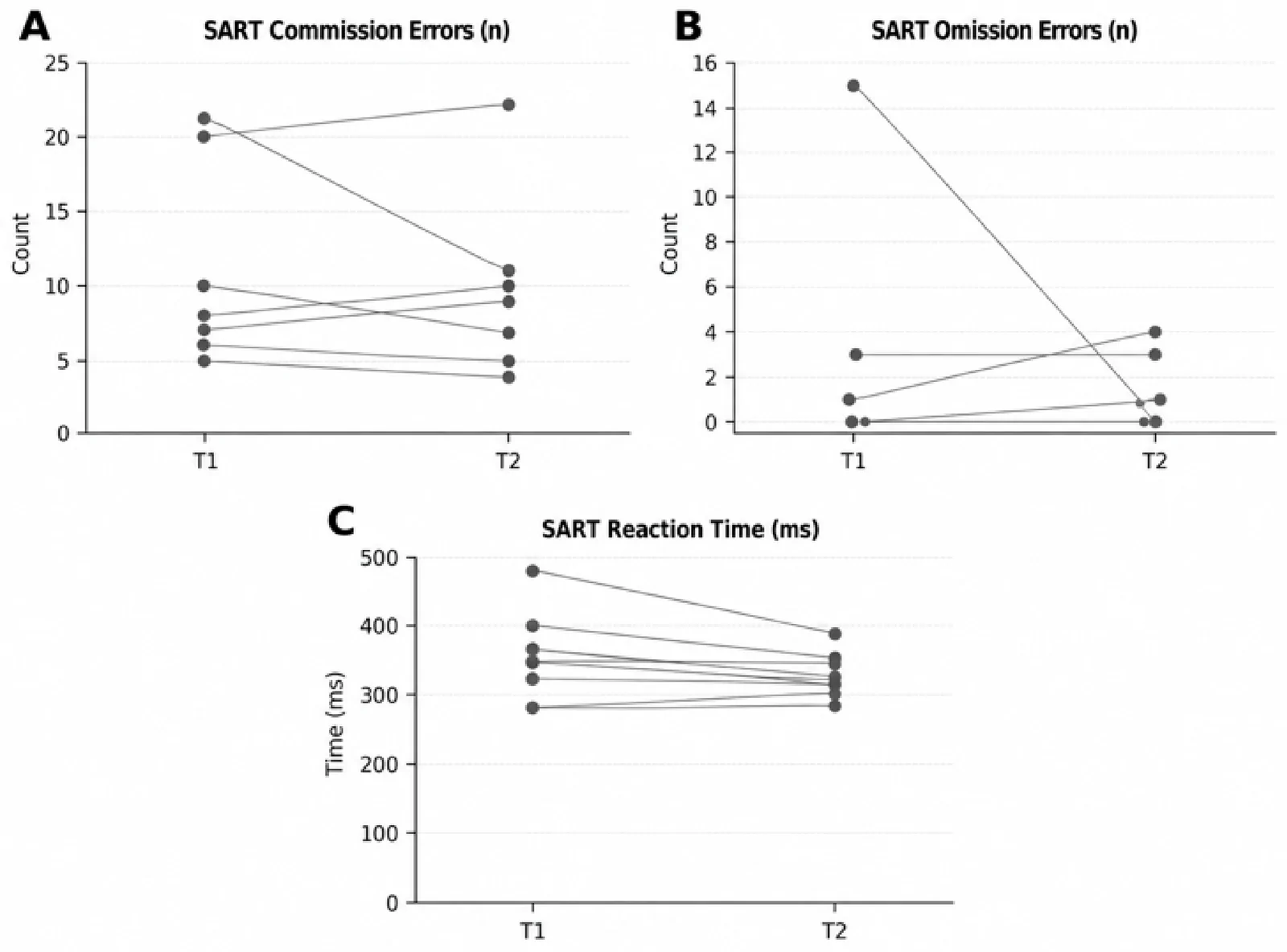
Individual Participant Changes in Sustained Attention to Response Task (SART) performance from baseline (T1) to post-intervention (T2): (**A**) Commision errors; (**B**) Omission errors; (**C**) Reaction Time. Lines connect paired observations for each participant.

**Figure 3:**
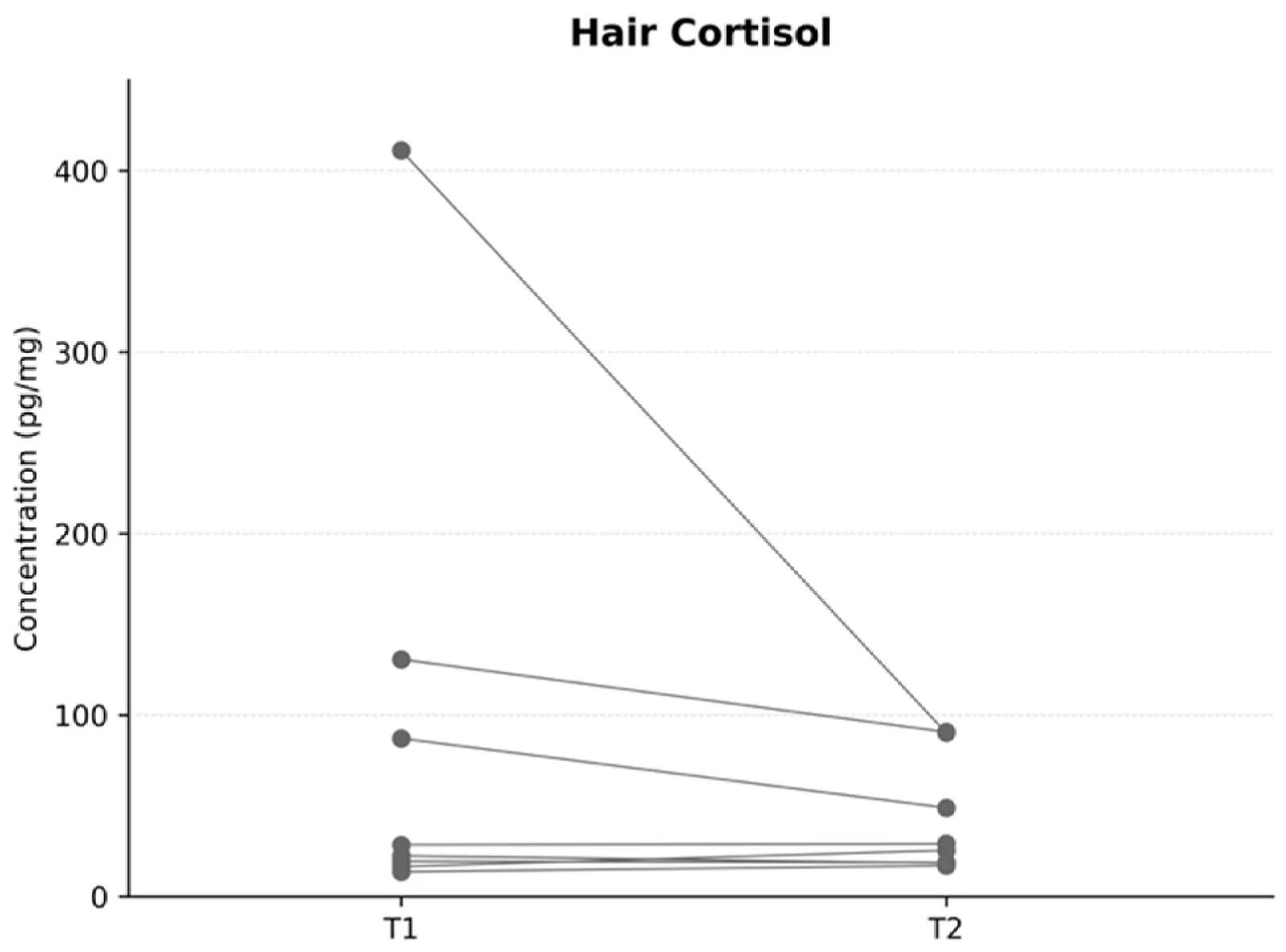
Individual Participant Changes in Hair Cortisol Concentration from T1 to T2.

Exploratory pre–post changes in participant-reported psychological measures, SART performance, and hair cortisol concentration are summarized in Table 2 and Figure 1, Figure 2 and Figure 3.

Positive affect increased from T1 to T2, with a mean paired change of +6.38 (95% CI, 0.96 to 11.79). Burnout, perceived stress, and PHQ-4 scores decreased, with mean paired changes of −0.49 (95% CI, −0.94 to −0.04), −3.50 (95% CI, −6.46 to −0.54), and −0.63 (95% CI, −2.29 to 1.04), respectively. For SART performance, mean paired changes were −0.63 commission errors (95% CI, −4.34 to 3.09), −1.13 omission errors (95% CI, −5.56 to 3.31), and −24.74 ms in reaction time (95% CI, −57.59 to 8.11).

The mean paired change in hair cortisol concentration was −48.88 pg/mg (95% CI, −142.06 to 44.30). Hair cortisol concentration and SART omission errors were right-skewed with influential observations. For this reason, T1 and T2 medians and interquartile ranges, along with median within-participant changes, are additionally presented for hair cortisol and SART omission errors in Table 2. In addition, standardized effect estimates and their 95% confidence intervals are presented in Table 2, and individual participant trajectories are shown in Figure 1, Figure 2 and Figure 3.

## 4. Discussion

The primary objective of this pilot study was to evaluate the feasibility and acceptability of implementing a faculty-delivered MBAT program within a PCCM fellowship. Feasibility was supported by successful delivery of all planned MBAT sessions, high session attendance, and complete study assessments and biospecimen collection, although independent mindfulness practice was limited both in frequency and duration. Acceptability was evaluated separately and was generally favorable based on participant ratings of motivation, application of MBAT skills to daily life, and willingness to recommend the course to colleagues. As secondary exploratory objectives, we examined pre–post changes in participant-reported psychological measures, objective attention, and hair cortisol concentration between T1 and T2. Observed changes included increased positive affect and decreases in burnout, perceived stress, PHQ-4 scores, SART error counts, reaction time, and hair cortisol concentration. Given the small, single-arm pilot design, these exploratory findings are intended to provide preliminary estimates rather than evidence of intervention efficacy.

Feasibility differed across the three domains evaluated. For intervention delivery and session attendance, all planned sessions were delivered, and attendance was high, although achieving this required substantial coordination and commitment from faculty instructors and fellowship leadership to provide protected time and accommodate fellows’ clinical schedules. Independent mindfulness practice represented a greater implementation challenge, with most participants practicing only 1–2 times per week, despite being asked to complete 15 min of independent mindfulness practice at least four days per week. Within the feasibility domains evaluated, sustaining independent mindfulness practice appeared to represent the greatest implementation challenge. Brief free-text responses identified difficulty remembering to practice, competing time demands, motivation or difficulty committing to practice, and fatigue related to clinical service as barriers. A strength of the study was the breadth and completeness of the data collection. All eight participants completed participant-reported psychological measures, a computerized objective attention task, and hair cortisol collection at both T1 and T2, resulting in no missing outcome data across this multimodal assessment battery. This supports the feasibility of incorporating psychological, behavioral, and biological measures within a future larger study of PCCM trainees. Future studies may consider investigating implementation strategies that promote habit formation, such as mobile applications incorporating guided exercises, practice tracking, and automated reminders, which may improve adherence to home practice and enhance the long-term sustainability of the intervention [30]. Relatedly, another line of work may be asynchronous, self-paced mindfulness training content. This approach may assist in reducing the substantial time commitment required by MBAT and other programs and may better accommodate participants’ demanding clinical and didactic schedules [31].

Although this study was conducted within PCCM fellowship, the implementation challenges identified—including the need for protected time, competing professional demands, fatigue, and difficulty sustaining independent practice—may also be relevant to other high-stress professional training environments. Future studies should examine whether faculty-delivered, train-the-trainer mindfulness models are feasible and acceptable across other disciplines while accounting for profession-specific demands and organizational contexts.

Participant-reported psychological measures, SART performance, and hair cortisol concentration were examined as exploratory secondary outcomes. Between T1 and T2, positive affect was higher while burnout, perceived stress, and anxiety and depression scores were lower. These patterns in participant-reported psychological measures are broadly consistent with previous studies reporting changes in physician well-being, resilience, and burnout following mindfulness-based interventions [32,33]. SART reaction time and both omission and commission errors were also lower at T2, while mean hair cortisol concentration decreased between T1 and T2. The confidence intervals around several of these exploratory estimates were broad, underscoring the uncertainty associated with estimates from this small pilot sample. Given the single-arm design and absence of a comparison condition, none of the observed pre–post changes can be attributed specifically to MBAT and may reflect temporal, contextual, or other uncontrolled influences. Interpretation of SART findings is further complicated by the potential for practice or familiarity effects with repeated administration. Similarly, interpretation of hair cortisol is limited by the absence of universally accepted normative reference values and the complex relationship between chronic stress, burnout, and hypothalamic–pituitary–adrenal axis regulation [34,35]. Nevertheless, the inclusion of participant-reported psychological measures, a performance-based measure of attention, and hair cortisol provided complementary perspectives on psychological, behavioral, and biological outcomes and can inform outcome selection and study design in future larger controlled evaluations of MBAT in physicians-in-training.

Several limitations should be acknowledged. Most importantly, the study included a small sample from a single institution and employed a single-arm pre-post design without a comparison group, limiting the ability to draw conclusions regarding intervention efficacy or generalize the findings to other training programs. The small sample size also precluded examining whether outcomes differed according to participants’ engagement in mindfulness practice outside of the scheduled MBAT sessions or by year of fellowship training. Future studies could focus on examining these variables and their relationship to study outcomes. Formal intervention fidelity was not prospectively assessed, limiting our ability to quantify adherence to the standardized curriculum or determine whether deviations occurred during intervention delivery. Future studies evaluating this faculty-delivered implementation model should incorporate structured fidelity monitoring and assessment of adherence to core intervention components. Because MBAT was delivered by faculty members within the participants’ fellowship program, hierarchical relationships and social-desirability bias may have influenced participants’ perceptions of the intervention, despite the use of privately completed, de-identified assessments and separation of intervention delivery from outcome assessment. Furthermore, adverse or unwanted effects related to mindfulness practice were not systematically assessed and should be evaluated in future studies. The small sample also resulted in wide confidence intervals around several exploratory pre-post estimates, limiting the precision of these findings and reinforcing their preliminary and hypothesis-generating nature. Additionally, the proximal 3 cm hair segments reflect approximately three months of cortisol exposure, resulting in substantially overlapping exposure windows for assessments conducted approximately five weeks apart; therefore, the pre–post comparison cannot isolate physiological changes occurring during the four-week MBAT intervention. Future studies should consider temporally non-overlapping hair-sampling windows or alternative biomarkers better aligned with the intervention duration. Because mindfulness training is intended to strengthen awareness and regulation of attention during everyday activities, future studies should complement objective task-based assessments with clinically embedded measures of attentional functioning at the bedside. Measures such as perceived focus during clinical tasks, attentional lapses, cognitive workload, and related patient-safety outcomes, including medical errors or near-miss events, may help determine whether the effects of MBAT translate into clinical practice [2].

## 5. Conclusions

In conclusion, this pilot study found favorable feasibility outcomes for intervention delivery and session attendance and for completion of planned study assessments and biospecimen collection within a PCCM fellowship, while independent mindfulness practice remained an important implementation challenge. The faculty-delivered, train-the-trainer MBAT program was also generally well accepted by participants. Exploratory analyses provided preliminary estimates of pre–post changes in participant-reported psychological measures, SART performance, and hair cortisol concentrations. As one of the first studies to evaluate MBAT in PCCM fellows, these findings contribute to the limited literature on mindfulness-based interventions in fellowship training and provide a foundation for future investigations. Further studies in larger cohorts using more rigorous study designs are needed to evaluate the effectiveness of this implementation model and its impact on physician well-being, stress, attention, and ultimately, patient care.

## Figures and Tables

**Table 1:** Feasibility and Acceptability of Faculty-Delivered Mindfulness-Based Attention Training (MBAT).

Feasibility and Acceptability Domains/Survey Items	Response	*n* (%)
Feasibility Domain 1: Intervention Delivery and Session Attendance		
Planned MBAT session offerings delivered	Delivered as intended	8 (100.0)
How many training sessions did you attend?	All 4 sessions	7 (87.5)
	3 of 4 sessions	1 (12.5)
List any obstacles to attending the training sessions.	No barrier reported	7 (87.5)
	Vacation/scheduling conflict	1 (12.5)
Feasibility Domain 2: Independent Mindfulness Practice		
On average, how many days did you practice per week?	Not at all	1 (12.5)
	1–2 times/week	6 (75.0)
	3–4 times/week	1 (12.5)
	≥5 times/week	0 (0.0)
When practicing, how long did you practice on average? †	<5 min	2 (28.6)
	5–10 min	2 (28.6)
	11–20 min	3 (42.9)
	>20 min	0 (0.0)
List any obstacles to practicing at home. ‡	No barrier reported	3 (37.5)
	Difficulty remembering to practice	4 (50.0)
	Limited time	2 (25.0)
	Motivation/commitment to practice	2 (25.0)
	Fatigue/exhaustion related to clinical service	1 (12.5)
How would you rate the overall quality of your mindfulness practices over the past month?	Very low	0 (0.0)
	Low	3 (37.5)
	Moderate	4 (50.0)
	High	1 (12.5)
	Very high	0 (0.0)
Feasibility Domain 3: Data Collection		
Completion of planned study assessments and biospecimen collection	Completed at T1 and T2	8 (100.0)
Acceptability: Participant perceptions		
I applied knowledge and skills from MBAT to my daily life.	Strongly disagree	0 (0.0)
	Disagree	1 (12.5)
	Neutral	1 (12.5)
	Agree	6 (75.0)
	Strongly agree	0 (0.0)
I was motivated to participate in the Mindfulness course.	Strongly disagree	0 (0.0)
	Disagree	1 (12.5)
	Neutral	2 (25.0)
	Agree	2 (25.0)
	Strongly agree	3 (37.5)
I would recommend that colleagues take a similar course.	Strongly disagree	0 (0.0)
	Disagree	1 (12.5)
	Neutral	0 (0.0)
	Agree	3 (37.5)
	Strongly agree	4 (50.0)

† Percentages are calculated among participants who reported independent mindfulness practice (*n* = 7). ‡ Participants could report more than one barrier; therefore, percentages may total more than 100%. Percentages are based on all eight participants.

**Table 2:** Exploratory Pre–Post Changes in Psychological Measures, Sustained Attention to Response Task (SART) Performance, and Hair Cortisol Concentration.

Outcome	T1 Mean ± SD	T2 Mean ± SD	Mean Paired Change (95% CI)	Cohen’s d_z_ (95% CI)
Self-reported psychological measures				
Positive Affect (PANAS)	30.0 ± 6.9	36.4 ± 3.9	+6.38 (0.96 to 11.79)	+0.98 (0.10 to 1.82)
Burnout (BAT-12)	2.91 ± 0.53	2.42 ± 0.49	−0.49 (−0.94 to −0.04)	−0.90 (−1.71 to −0.05)
Perceived Stress (PSS)	19.5 ± 5.8	16.0 ± 5.9	−3.50 (−6.46 to −0.54)	−0.99 (−1.82 to −0.11)
Anxiety/Depression (PHQ-4)	2.63 ± 1.92	2.00 ± 1.20	−0.63 (−2.29 to 1.04)	−0.31 (−1.01 to 0.41)
SART				
SART Reaction Time (ms)	352.0 ± 65.9	327.3 ± 32.6	−24.74 (−57.59 to 8.11)	−0.63 (−1.38 to 0.15)
SART Omission Errors (*n*)	2.38 ± 5.21	1.25 ± 1.49	−1.13 (−5.56 to 3.31)	−0.21 (−0.91 to 0.50)
Median (Q1–Q3) †	0 (0–1.50)	1 (0–1.50)	0 (median of paired changes)	—
SART Commission Errors (*n*)	10.38 ± 6.44	9.75 ± 5.55	−0.63 (−4.34 to 3.09)	−0.14 (−0.83 to 0.56)
Cortisol				
Hair Cortisol (µg/dL)	91.17 ± 135.92	42.29 ± 31.44	−48.88 (−142.06 to 44.30)	−0.44 (−1.15 to 0.30)
Median (Q1–Q3) †	25.44 (18.68–98.05)	27.21 (18.63–59.21)	−2.37 (median of paired changes)	—

Mean paired changes are T2–T1. CI = confidence interval; SD = standard deviation. † Additional rows report T1 and T2 medians (Q1–Q3); the change column reports the median within-participant change. PANAS = Positive Affect Schedule; BAT-12 = Burnout Assessment Tool; PSS = Perceived Stress Scale; PHQ-4 = Patient Health Questionnaire-4; SART = Sustained Attention to Response Task.

## Data Availability

The raw data supporting the conclusions of this article will be made available by the authors on request. The individual-level data are not available due to the risk of participant re-identification in this small, institution-specific cohort.
